# p14 expression differences in ovarian benign, borderline and malignant epithelial tumors

**DOI:** 10.1186/s13048-016-0275-2

**Published:** 2016-10-22

**Authors:** Vinicius Duarte Cabral, Marcelle Reesink Cerski, Ivana Trindade Sa Brito, Lucia Maria Kliemann

**Affiliations:** Serviço de Patologia, Hospital de Clínicas de Porto Alegre, Universidade Federal do Rio Grande do Sul, Rua Ramiro Barcelos 2350, Porto Alegre, RS 90035-90 Brazil

**Keywords:** Ovary, Ovarian epithelial tumor, Cancer, p14, ARF, p16, p53, Immunohistochemistry

## Abstract

**Background:**

Abnormalities in tumor suppressors p14, p16 and p53 are reported in several human cancers. In ovarian epithelial carcinogenesis, p16 and p53 show higher immunohistochemical staining frequencies in malignant tumors and are associated with poor prognoses. p14 was only analyzed in carcinomas, with conflicting results. There are no reports on its expression in benign and borderline tumors. This study aims to determine p14, p16 and p53 expression frequencies in ovarian benign, borderline and malignant tumors and their associations with clinical parameters.

**Methods:**

A cross-sectional study utilizing immunohistochemistry was performed on paraffin-embedded ovarian epithelial tumor samples. Clinical data were collected from medical records. Fisher’s exact test and the Bonferroni correction were performed for frequency associations. Survival comparisons utilized Kaplan-Meier and log rank testing. Associations were considered significant when *p* < 0.05.

**Results:**

p14 absent expression was associated with malignant tumors (60 % positive) (*p* = 0.000), while 93 % and 94 % of benign and borderline tumors, respectively, were positive. p16 was positive in 94.6 % of carcinomas, 75 % of borderline and 45.7 % of benign tumors (*p* = 0.000). p53 negative staining was associated with benign tumors (2.9 % positive) (*p* = 0.016) but no difference was observed between borderline (16.7 %) and malignant tumors (29.7 %) (*p* = 0.560). No associations were found between expression rates, disease-free survival times or clinical variables. Carcinoma subtypes showed no difference in expression.

**Conclusions:**

This is the first description of p14 expression in benign and borderline tumors. It remains stable in benign and borderline tumors, while carcinomas show a significant absence of staining. This may indicate that p14 abnormalities occur later in carcinogenesis. p16 and p53 frequencies increase from benign to borderline and malignant tumors, similarly to previous reports, possibly reflecting the accumulation of inactive mutant protein. The small sample size may have prevented statistically significant survival analyses and clinical correlations. Future studies should investigate genetic abnormalities in p14 coding sequences and include all types of ovarian epithelial tumors. Bigger sample sizes may be needed for significant associations.

## Background

Ovarian cancer has the seventh highest cancer incidence in women worldwide. In 2012, 238,719 new cases were diagnosed and 151,917 women died from the disease [1]. Malignant epithelial tumors comprise 90 % of all ovarian cancers and are usually diagnosed at advanced stages, leading to high mortality and low survival rates [2, 3]. Abnormalities in cell cycle control have been reported in a wide range of human cancers [4, 5]. The eukaryotic cell cycle is divided in five phases: mitosis, gap 0 (G0) gap 1 (G1), synthesis (S) and gap 2 (G2). Most human adult cells are in G0, with no increase in cell size or DNA content. This state is maintained by tumor suppressor protein pRb. When external growth factors are present, the cell enters the G1 phase, during which there is duplication of all cellular components, except DNA. This is possible due to the release of the suppressing effects of pRb through the action of complexes formed by cyclins and cyclin-dependent kinases. The inactivation of pRb allows progression through G1 and into the synthesis phase, when DNA is replicated. When DNA damage, insufficient cell size or oncogenic stimuli are present, other tumor suppressor proteins are activated and prevent passage from G1 to S. If abnormalities are present in these proteins, mutated cells can proliferate unrestrictedly and generate a neoplasm [6–8]. Tumor suppressors’ genetic and immunohistochemical alterations are a common finding in human cancers, including ovarian epithelial tumors, specially carcinomas [9–16]. In this study, we focus on three of these proteins: p14, p16 and p53.

p14 (also known as ARF) is transcribed from the CDKN2A gene. It sequesters MDM2 in the nucleolus, preventing p53 degradation. Additionally, it inhibits transcription factor E2F activity. These actions lead to cell cycle arrest [17, 18]. Previous studies about p14 immunohistochemical expression in ovarian epithelial tumors have focused only on carcinomas and display conflicting results. Saegusa et al. described positivity in all carcinomas [19]. Hashiguchi et al. reported positivity in 89.1 % of all carcinomas, while Khouja et al. found that 89 % of carcinomas were negative [20, 21]. None of these studies demonstrated any association with clinical parameters or prognosis. We found no reports on p14 expression in benign and borderline tumors.

p16 is also a product of CDKN2A. It prevents cell cycle advance by inhibiting cyclin D-cdk4/6, thus maintaining pRb’s negative control over the restriction point [10]. p16 is suppressed by pRb, and high expression of p16 is associated with loss of pRb function, one of the most common abnormalities in cancer [22]. Higher expression rates are found in carcinomas, followed by borderline and benign tumors, despite a wide staining percentage variation among studies [15, 23]. Some authors report differences between high-grade serous carcinoma (HGSC) and low-grade serous carcinoma (LGSC), with the latter not differing from serous borderline tumors. However, other studies show no difference between HGSC and LGSC [16, 24–27]. When evaluated as prognostic factors, both high and low levels of p16 staining have been linked to worse outcomes, while intermediate levels were associated with longer survival times. Other reports found no association with survival [23, 25–27]. In relation to epithelial types, serous tumors show high expression, while mucinous and endometrioid tumors demonstrate low levels or absent staining [25, 28, 29].

p53 can arrest cell cycle progression or activate the apoptosis pathway if DNA damage is extensive. In unaltered cells, p53 is constantly degraded by MDM2, leading to a very short half-life. When DNA damage is present, several factors (such as p14) cause MDM2 phosphorylation. p53 is stabilized and cellular levels rise, serving as a transcription factor for some genes and repressing others [6]. Mutations in TP53 are a common event in most human cancers, transcribing inactive proteins, which are resistant to degradation and detectable by immunohistochemistry [22]. Ovarian carcinomas exhibit the highest rates of expression in comparison to borderline and benign tumors. Among carcinoma subtypes, HGSC are more often positive than LGSC, mucinous, endometrioid and clear cell carcinomas [15, 30, 31]. O’Neill et al. reported a high level of expression in 64 % of HGSC and 18 % of LGSC [24]. Some reports indicated that high levels of expression relate to poor prognosis, but others found no association [15, 30]. In this study, we aimed to assess p14, p16 and p53 expression in ovarian epithelial tumors and to determine associations with disease-free survival time and clinical variables.

## Results

**Fig. 1:**
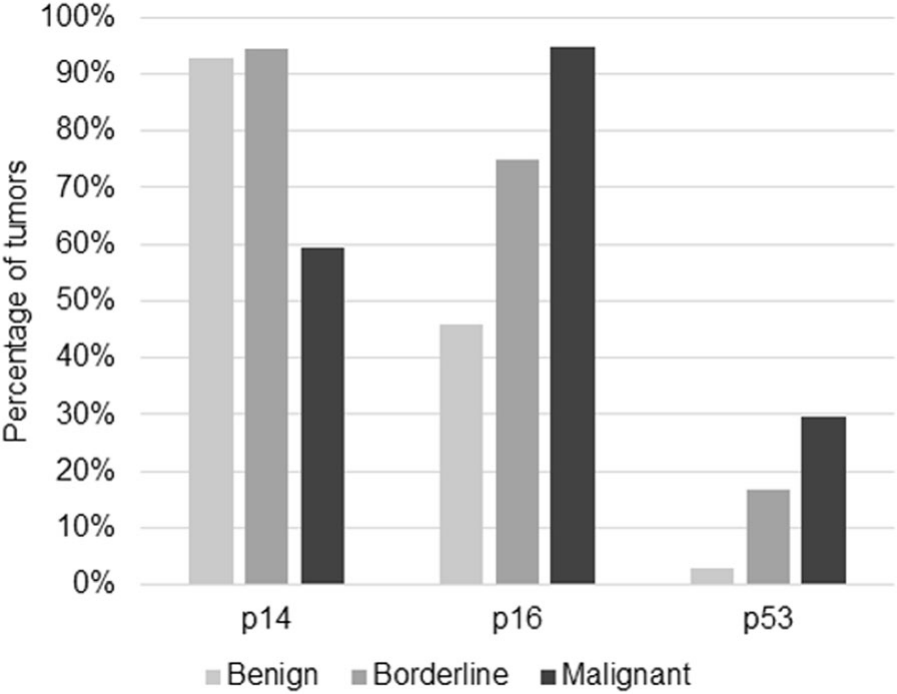
Percentage of p14, p16 and p53 positive tumors in benign, borderline and malignant tumors

Mean age of diagnosis was 49.36 (45.68–53.03) for benign tumors, 44.25 (38.24–50.26) for borderline tumors and 58.35 (54.39–62.32) for malignant tumors (95 % confidence interval). Malignant tumors were associated with older age, while benign and borderline tumors showed no statistical difference (*p* = 0.048). FIGO stage information was available in 17.5 % of cases and no statistically significant associations were found. The highest rates of p14 immunoreactivity were found in benign tumors, decreasing in borderline tumors and even further in carcinomas, as shown in Table 1. When considered positive (score 1 or higher) or negative, 93 % of benign, 94 % of borderline and 60 % of malignant tumors were positive (Fig. 1). Absent expression was associated with carcinomas when compared to borderline and benign tumors (*p* = 0.000), but no difference was observed between the last two (*p* = 1.000). Serous and mucinous tumors demonstrated no difference in expression (85.1 % and 89.7 %, respectively) (*p* = 1.000). Endometrioid tumors were significantly associated with negative expression (all tumors were negative) (*p* = 0.000).

**Table 1:** p14 expression in benign, borderline and malignant tumors

PPN(^a^)	Benign	Borderline	Malignant
0	7.1 %	5.6 %	40.5 %
1–10	5.7 %	25 %	18.9 %
11–50	47.1 %	66.7 %	35.1 %
51–100	40 %	2.8 %	5.4 %

(^a^) Percentage of positive nuclei

p16 expression was higher in malignant tumors, decreasing in borderline and benign tumors (Table 2). No borderline or malignant tumor showed less than 11 % positive nuclei. Positivity (score 3) was found in 94.6 % of carcinomas, 75 % of borderline and 45.7 % of benign tumors (Fig. 1). The different rates among all three types were statistically significant (*p* = 0.000). 71.3 % of serous, 48.7 % of mucinous and 100 % of endometrioid tumors were positive, but only serous and mucinous tumors were statistically different (*p* = 0.034).

**Table 2:** p16 expression in benign, borderline and malignant tumors

PPN (^a^)	Benign	Borderline	Malignant
0	1.4 %	0 %	0 %
1–10	14.4 %	0 %	0 %
11–50	38.6 %	25 %	5.4 %
51–100	45.7 %	75 %	94.6 %

(^a^) Percentage of positive nuclei

p53 staining was higher in malignant tumors, decreasing in borderline and in benign tumors. No benign or borderline tumor exhibited more than 50 % positive nuclei (Table 3). 29.7 % of malignant, 16.7 % of borderline and 2.9 % of benign tumors were positive (score 2 or 3) (Fig. 1). The rate difference was significant between benign and borderline (*p* = 0.016), benign and malignant tumors (*p* = 0.000), but not between borderline and malignant tumors (*p* = 0.560). 66.7 % of endometrioid, 12.8 % of mucinous and 11.9 % of serous tumors were positive. Statistical significance was demonstrated between endometrioid and serous tumors (*p* = 0.017). No antibody showed expression differences among carcinoma subtypes. No correlations were found when comparing expression rates between each other. We did not encounter associations with disease-free survival times or other clinical parameters.

**Table 3:** p53 expression in benign, borderline and malignant tumors

PPN (^a^)	Benign	Borderline	Malignant
0	92.9 %	63.9 %	64.9 %
1–10	4.3 %	19.4 %	5.4 %
11–50	2.9 %	16.7 %	18.9 %
51–100	0 %	0 %	10.8 %

(^a^) Percentage of positive nuclei

## Discussion

The main limitation of the study was its small sample size. We believe this prevented statistically significant associations between immunohistochemical expression and disease-free survival and clinical data. Our main finding was the determination of p14 levels in benign and borderline tumors and their difference in relation to carcinomas. To our knowledge, no other study has investigated p14 in these tumors. 93 % and 94 % of benign and borderline tumors, respectively, were positive. Carcinomas showed a significant absent expression, as reported by Khouja et al. and Havrilesky et al. [21, 22]. Khouja et al. also reported no expression in normal ovaries (*n* = 10). The high expression levels in benign and borderline tumors may indicate wild-type p14 is not detectable, with p14 mutation occurring early in ovarian carcinogenesis. This would lead to mutant proteins, unresponsive to negative feedback mechanisms, detectable by immunohistochemistry. In laryngeal cancer, normal tissue shows low levels (25 %), while dysplastic (82 %) and neoplastic (100 %) tissues are mostly positive [32, 33]. Subsequent mutations could lead to loss of function and a corresponding diminished expression in carcinomas. Another possible explanation for the progressive increase in p14 expression is that its activity is maintained throughout carcinogenesis, being overexpressed in response to oncogenic signals. A mutation, loss of function and expression would be late events. This has been proposed in endometrial carcinogenesis, where adenocarcinomas show greater expression than normal endometrium, but high-grade tumors are associated with absent staining [12]. In renal cell carcinoma, El-Mokadem et al. [34] reported lower expression in neoplastic tissue when compared to normal kidney samples.

p16 levels were similar to previous reports: carcinomas are the most positive, followed by borderline tumors and adenomas [23, 24]. 94.6 % of carcinomas were positive, comparable to reports by Dong et al. and Kommoss et al. [23, 26]. There is no consensus on interpretation methods or cutoff values for positivity, leading to a wide range of expression rates. Different clones also influence this variation [21, 25]. The progressive increase in expression was also reported in normal mammary tissue and breast carcinomas [13]. Possible explanations include: progressive loss of pRb function and its repressive effect over p16; bypassing of p16’s tumor suppressing effect by alterations in other components of cell cycle control, leading to overexpression in an attempt to maintain the control over cellular proliferation; accumulation of inactive mutant proteins [10, 14, 22, 35]. We could not find differences in expression among carcinoma subtypes. However, p16 levels were statistically different between carcinomas and borderline tumors. Given that most carcinomas were high grade, this finding may corroborate the assumption that HGSC pathogenesis is independent from benign and borderline tumors [35]. High p16 levels were associated with serous histology, as previously described by Dong et al. and O’Neill et al. [23, 24]. There was no association between p16 and p14 levels. Despite being transcribed from the same locus, they differ in exon 1. Mutations and methylations were reported in their shared and unique sequences, explaining why they display non-related expressions [11, 20, 36, 37].

p53 expression increased from benign to borderline and malignant tumors, as previously reported [30]. Analogously to p14 and p16, reports on p53 expression have different designs, interpretation methods and definitions of positive or negative. Expression rates vary according to the clone utilized. As reported by Kmet et al. [30], the most commonly used clones show a prevalence of more than 50 % positivity in carcinomas. In our sample, 29.7 % of carcinomas were positive, a similar result to another study using the same antibody (PAb240). The gradual increase in p53 expression is explained by different half-lives between the wild-type protein (constantly degraded, short half-life) and mutant p53 (resistant to degradation, long half-life). While the former is rarely detectable, the latter accumulates in the cell and is more likely to be detected immunohistochemically [9]. It has been proposed that wild-type p53 inhibits p16 expression. Despite not showing statistical association, both staining percentages increased in a similar manner, which is compatible with this theory [21, 22]. The majority of authors report significant differences between HGSC and LGSC or serous borderline tumors [19, 31]. We did not find a statistically significant difference between borderline and malignant tumors or among carcinoma types. Endometrioid tumors were statistically associated with negative p14 (*p* = 0.04) and positive p53 (*p* = 0.047). While *p* < 0.05, we do not believe this is a valid association, given that all our endometrioid sample was composed of carcinomas, which are associated with low p14 and high p53 staining.

## Conclusions

We have demonstrated that p14 is positive in benign and borderline tumors, while absent staining is associated with carcinomas. As observed in other organs, this may indicate that loss of p14 activity is a late event in tumorigenesis [33, 38]. Expression in normal ovaries has been reported as absent, but this was based on a small (*n* = 10) sample [21]. Further studies are necessary to determine staining levels in non-neoplastic ovarian tissue. We did not find associations between staining indexes and disease-free survival times or clinical parameters. Even though previous studies showed no prognostic relevance, we believe larger samples are necessary to determine if p14 has any unreported associations. p14 is not as extensively studied in ovarian tumors as p16, and reports on mutations and methylations of CDKN2A rarely specify if these abnormalities affect their shared or individual exons [11, 20, 36, 37]. Future studies should report not only the presence of these alterations, but also their location in CDKN2A. Additionally, they should include benign and borderline tumors.

## Methods

### Sample selection

This is a cross-sectional study using buffered, 10 % formalin-fixed, paraffin-embedded tissue samples from women who underwent surgery for primary ovarian epithelial tumors at Hospital de Clinicas de Porto Alegre between January 2007 and January 2014. Clinical information (age, FIGO stage, surgical findings, oral contraceptive use, parity and family history of ovarian or breast cancer) and disease-free times (defined as the number of months from diagnosis to recurrence or death) were collected from medical records. The original hematoxylin and eosin stained slides were reviewed and one paraffin block was selected from each case. We excluded possible metastatic tumors, samples with insufficient epithelium for immunohistochemical staining and those with undifferentiated histology. Brenner tumors were not included due to their rarity in our archives. The final sample contained 143 tumors: 47 serous cystadenomas, 23 mucinous cystadenomas, 24 serous borderline tumors, 12 mucinous borderline tumors, 4 low-grade serous carcinomas, 25 high-grade serous carcinomas, 5 mucinous carcinomas and 3 endometrioid carcinomas.

### Immunohistochemistry

Whole tissue sections were cut 3 μm thick and mounted on silanized slides. Slides were incubated at 80 °C for 60 min, depariffinized and rehydrated. Water-bath antigen retrieval was performed at 95 °C for 60 min, utilizing citrate buffer ph 6.0. Endogenous peroxidase activity was blocked with 3 % hydrogen peroxide in methanol solution for 30 min. Slides were incubated overnight at 4 °C with the following primary antibodies: anti-p14 (1:25, ab3642, abcam®), anti-p16 (1:400, ab54210, abcam®) and anti-p53 (1:100, ab26, abcam®). ADVANCE™ (Dako) was used for secondary antibody incubation, followed by hematoxylin counterstaining and mounting. Positive and negative controls for each antibody were also stained.

**Fig. 2:**
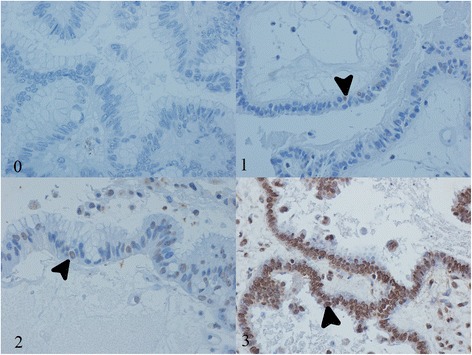
Expression scores. Immunohistochemical scores: 0 (0 % positive nuclei), 1 (1–10 % positive nuclei), 2 (11–50 %) positive nuclei, 3 (51–100 % positive nuclei). Arrows indicate positive nuclei

Immunohistochemical expression was assessed individually by two pathologists. A third pathologist reviewed conflicting results and a final score was reached. Due to excessive background staining and previous reports about the lack of specificity of cytoplasmic expression, only unequivocal nuclear positivity was considered valid [19]. Each tissue section was read in its entirety and a score was assigned according to the percentage of positive nuclei in neoplastic epithelial cells: 0 (0 %), 1 (1–10 %), 2 (11–50 %) or 3 (51–100 %) (Fig. 2). Based on previous studies, for p14, scores 1 or greater were considered positive [21]. For p16, only score 3 and for p53, scores 2 and 3 were taken as positive [16].

### Statistical analysis

IBM SPSS Statistics version 22.0 (IBM Corporation, 2013) was used for all statistical calculations. Expression of p14, p16 and p53 and their association with other parameters were evaluated by Fisher’s exact test and the Bonferroni correction. For disease-free survival analyses, we used Kaplan-Meier’s estimate and the log-rank test. 95 % confidence interval for the mean age of patients at diagnosis was submitted to post-hoc analysis (ANOVA and Tukey’s range test). Associations were considered statistically significant when *p* < 0.05.

## Abbreviations

G0: Gap 0
G1: Gap 1
G2: Gap 2
HGSC: High-grade serous carcinoma
LGSC: Low-grade serous carcinoma
PPN: Percentage of positive nuclei
S: Synthesis

## References

[1] Ferlay J, Soerjomataram I, Ervik M, Dikshit R, Eser S, Mathers C, Rebelo M, Parkin DM, Forman D, Bray F. GLOBOCAN 2012 v1.1, Cancer Incidence and Mortality Worldwide: IARC CancerBase No. 11 [Internet]. Lyon, Fr. Int Agency Res Cancer. 2014. [cited 2016 Feb 13]. Available from: http://globocan.iarc.fr. Accessed 13 Feb 2016.

[2] Trétarre B, Molinie F, Woronoff A-S, Bossard N, Bessaoud F, Marrer E, et al. Ovarian cancer in France: Trends in incidence, mortality and survival, 1980–2012. Gynecol Oncol. 2015;139:324–9. Elsevier Inc; Available from: http://www.sciencedirect.com/science/article/pii/S009082581530130X. 10.1016/j.ygyno.2015.09.013 26383829

[3] Lowe K, Chia VM, Taylor A, O’Malley C, Kelsh M, Mohamed M (2013). An international assessment of ovarian cancer incidence and mortality. Gynecol Oncol.

[4] Williams GH, Stoeber K (2012). The cell cycle and cancer. J Pathol.

[5] Wang H, Zhang X, Teng L, Legerski RJ (2015). DNA damage checkpoint recovery and cancer development. Exp Cell Res.

[6] Krebs JE, Goldstein ES, Kilpatrick ST. Lewin’s Genes XI [Internet]. 2014. Available from: http://books.google.com/books?id=yXFfPkLq4yEC&pgis=1. Accessed 13 Feb 2016.

[7] Cooper GM, Hausman RE (2013). The Cell: A Molecular Approach.

[8] Malumbres M (2011). Physiological Relevance of Cell Cycle Kinases. Physiol Rev.

[9] Brosh R, Rotter V (2009). When mutants gain new powers: news from the mutant p53 field. Nat Rev Cancer.

[10] Li J, Poi M, Tsai M. Regulatory mechanisms of tumor suppressor P16INK4A and their relevance to cancer. Biochemistry. 2011;5566–82. [cited 2016 Feb 13]. Available from: http://pubs.acs.org/doi/abs/10.1021/bi200642e. 10.1021/bi200642e PMC3127263 21619050

[11] Boström J, Meyer-Puttlitz B, Wolter M, Blaschke B, Weber RG, Lichter P (2001). Alterations of the tumor suppressor genes CDKN2A (p16(INK4a)), p14(ARF), CDKN2B (p15(INK4b)), and CDKN2C (p18(INK4c)) in atypical and anaplastic meningiomas. Am J Pathol.

[12] Watanabe J, Nishizaki R, Jobo T, Kamata Y, Hata H, Nishimura Y (2004). Expression of tumor suppressor gene product p14ARF in endometrioid adenocarcinoma of the uterine corpus. Int J Gynecol Pathol.

[13] Pare R, Shin J-S, Lee SC. Increased expression of senescence markers p14 ^ARF^ and p16 ^INK^^4a^ in breast cancer is associated with increased risk of disease recurrence and poor survival outcome. Histopathology. 2016. Available from: http://doi.wiley.com/10.1111/his.12948. 10.1111/his.12948 26843058

[14] Saldanha SN, Tollefsbol TO. Pathway modulations and epigenetic alterations in ovarian tumorbiogenesis. J Cell Physiol. 2014;229:393–406. 10.1002/jcp.24466 PMC3875779 24105793

[15] Nam EJ, Kim YT (2008). Alteration of cell-cycle regulation in epithelial ovarian cancer. Int J Gynecol Cancer.

[16] Lee YH, Heo J, Kim TH, Kang H, Kim G, Kim J (2011). Significance of cell cycle regulatory proteins as malignant and prognostic biomarkers in ovarian epithelial tumors. Int J Gynecol Pathol.

[17] Mason SL, Loughran O, La Thangue NB (2002). p14(ARF) regulates E2F activity. Oncogene.

[18] Sherr CJ (2006). Divorcing ARF and p53: an unsettled case. Nat Rev Cancer.

[19] Machida D, Ph BD, Okayasu I, Saegusa M, Machida D, Okayasu I (2001). Possible Associations among Expression of p14 ARF, and the Balance of Apoptosis and Cell Proliferation in Ovarian Carcinomas. Cancer.

[20] Hashiguchi Y, Tsuda H, Yamamoto K, Inoue T, Ishiko O, Ogita S (2001). Combined Analysis of p53 and RB Pathways in Epithelial Ovarian Cancer. Hum Pathol.

[21] Khouja MH, Baekelandt M, Nesland JM, Holm R (2007). The clinical importance of Ki-67, p16, p14, and p57 expression in patients with advanced ovarian carcinoma. Int J Gynecol Pathol.

[22] Havrilesky L, Darcy Kathleen M, Hamdan H, Priore RL, Leon J, Bell J (2003). Prognostic significance of p53 mutation and p53 overexpression in advanced epithelial ovarian cancer: a Gynecologic Oncology Group Study. J Clin Oncol.

[23] Dong Y, Walsh MD, McGuckin MA, Gabrielli BG, Cummings MC, Wright RG (1997). Increased expression of cyclin-dependent kinase inhibitor 2 (CDKN2A) gene product P16INK4A in ovarian cancer is associated with progression and unfavourable prognosis. Int J Cancer.

[24] O’Neill CJ, McBride HA, Connolly LE, Deavers MT, Malpica A, McCluggage WG (2007). High-grade ovarian serous carcinoma exhibits significantly higher p16 expression than low-grade serous carcinoma and serous borderline tumour. Histopathology.

[25] Felix AS, Sherman ME, Hewitt SM, Gunja MZ, Yang HP, Cora RL (2015). Cell-Cycle Protein Expression in a Population-Based Study of Ovarian and Endometrial Cancers. Front Oncol.

[26] Kommoss S, du Bois A, Ridder R, Trunk MJ, Schmidt D, Pfisterer J (2007). Independent prognostic significance of cell cycle regulator proteins p16(INK4a) and pRb in advanced-stage ovarian carcinoma including optimally debulked patients: a translational research subprotocol of a randomised study of the Arbeitsgemeinschaft Gynaek. Br J Cancer.

[27] Milde-Langosch K, Hagen M, Bamberger A-M, Löning T (2003). Expression and prognostic value of the cell-cycle regulatory proteins, Rb, p16MTS1, p21WAF1, p27KIP1, cyclin E, and cyclin D2, in ovarian cancer. Int J Gynecol Pathol.

[28] Vang R, Shih I, Kurman R (2009). Ovarian low-grade and high-grade serous carcinoma: pathogenesis, clinicopathologic and molecular biologic features, and diagnostic problems. Adv Anat Pathol.

[29] Vang R, Gown AM, Farinola M, Barry TS, Wheeler DT, Yemelyanova A (2007). p16 expression in primary ovarian mucinous and endometrioid tumors and metastatic adenocarcinomas in the ovary: utility for identification of metastatic HPV-related endocervical adenocarcinomas. Am J Surg Pathol.

[30] Kmet LM, Cook LS, Magliocco AM (2003). A review of p53 expression and mutation in human benign, low malignant potential, and invasive epithelial ovarian tumors. Cancer.

[31] Giurgea LN, Ungureanu C, Mihailovici MS (2012). The immunohistochemical expression of p53 and Ki67 in ovarian epithelialborderline tumors. Correlation with clinicopathological factors. Rom J Morphol Embryol.

[32] Bai P, Xiao X, Zou J, Cui L, Bui Nguyen TM, Liu J (2012). Expression of p14(ARF), p15(INK4b), p16(INK4a) and skp2 increases during esophageal squamous cell cancer progression. Exp Ther Med.

[33] Li Z, Ding S, Zhong Q, Li G, Zhang Y, Chen X, et al. Significance of MMP11 and P14 ARF expressions in clinical outcomes of patients with laryngeal cancer. Int J Clin Exp Med. 2015;8:15581–90. PMC4658941 26629052

[34] El-Mokadem I, Lim A, Kidd T, Garret K, Pratt N, Batty D (2016). Microsatellite alteration and immunohistochemical expression profile of chromosome 9p21 in patients with sporadic renal cell carcinoma following surgical resection. BMC Cancer.

[35] Milea A, George SH, Matevski D, Jiang H, Madunic M, Berman HK (2013). Retinoblastoma pathway deregulatory mechanisms determine clinical outcome in high-grade serous ovarian carcinoma. Mod Pathol.

[36] Esteller M, Tortola S, Toyota M, Capella G, Peinado MA, Baylin SB, et al. Hypermethylation-associated Inactivation of p14 ARF Is Independent of p16 INK4a Methylation and p53 Mutational Status 1. Cancer Res. 2000;60(1):129–133. 10646864

[37] Brown VL, Harwood CA, Crook T, Cronin JG, Kelsell DR, Proby CM (2004). p16INK4a and p14ARF tumor suppressor genes are commonly inactivated in cutaneous squamous cell carcinoma. J Invest Dermatol.

[38] Watanabe J, Nishizaki R, Jobo T, Kamata Y, Hata H, Nishimura Y (2004). Molecular profiling uncovers a p53-associated role for microRNA-31 in inhibiting the proliferation of serous ovarian carcinomas and other cancers. Oncogene.

